# Estimate of the energy value of soybean meal relative to corn based on growth performance of nursery pigs

**DOI:** 10.1186/s40104-020-00474-x

**Published:** 2020-07-03

**Authors:** Henrique S. Cemin, Hayden E. Williams, Mike D. Tokach, Steve S. Dritz, Jason C. Woodworth, Joel M. DeRouchey, Robert D. Goodband, Kyle F. Coble, Brittany A. Carrender, Mandy J. Gerhart

**Affiliations:** 1 Department of Animal Sciences and Industry, College of Agriculture, Kansas State University, Manhattan, KS 66506 USA; 2 Department of Diagnostic Medicine/Pathobiology, College of Veterinary Medicine, Kansas State University, Manhattan, KS 66506 USA; 3 JBS USA, Greeley, CO 80634 USA

**Keywords:** Caloric efficiency, Energy, Soybean meal, Swine

## Abstract

**Background:**

Two experiments were conducted to determine the effects of increasing amounts of soybean meal (SBM) in swine diets and estimate the energy value of SBM.

**Methods:**

A total of 2233 pigs (PIC 337 × 1050, Hendersonville, TN) and 3796 pigs (PIC 359 × C40), initially 11.0 kg and 17.6 kg body weight (BW), were used in Exp. 1 and 2, respectively. In Exp. 1, pigs were placed in 92 pens each containing 20 to 27 pigs. In Exp. 2, pigs were placed in 84 pens each containing 37 to 43 pigs. Treatments were assigned in a randomized complete block design with BW as the blocking factor. Dietary treatments consisted of 21%, 27%, 33%, or 39% SBM in Exp. 1 and 17.5%, 22%, 26.5%, 31%, 35.5%, or 40% SBM in Exp. 2, obtained by changing the inclusion rate of feed-grade amino acids and corn grain. For Exp. 1, representative samples of corn grain, SBM, and distillers dried grains with solubles were analyzed for total AA content prior to diet formulation. For Exp. 2, diets were formulated using NRC (2012) nutrient loadings. Treatment diets were fed for 21 and 22 d (Exp. 1 and 2) and there were 23 replicates in Exp. 1 and 14 replicates in Exp. 2. Pigs were weighed and feed disappearance measured weekly to calculate average daily gain (ADG), average daily feed intake (ADFI), gain-to-feed ratio (G:F), and caloric efficiency (CE). Data were analyzed with block as a random effect and treatment as a fixed effect, and contrasts were constructed to test the linear and quadratic effects of increasing SBM.

**Results:**

In Exp. 1, there was a tendency (linear, *P* = 0.092) for a decrease in ADFI as SBM increased. There was a tendency (*P* = 0.090) for a quadratic response for ADG, with a decrease in ADG observed with 39% SBM inclusion. Pigs fed diets with increasing SBM had a tendency (quadratic, *P* = 0.069) for an increase in G:F up to 33% SBM and an improvement (linear, *P* = 0.001; quadratic, *P* = 0.063) in CE with increasing SBM. Using CE to estimate the energy of SBM relative to corn, a value of 105.4% of corn energy or 2816 kcal/kg NE was determined using all data points. When removing the CE value of the 39% SBM treatment due to the quadratic tendency, SBM was estimated to have 121.1% of corn energy or 3236 kcal/kg NE. In Exp. 2, there was a decrease (linear, *P* = 0.001) in ADFI. Pigs fed increasing SBM had a tendency (linear, *P* = 0.065) for reduced ADG but an improvement (linear, *P* = 0.001) in G:F and CE as SBM increased. The energy value of SBM was estimated as 124.7% of corn energy or 3332 kcal/kg NE.

**Conclusions:**

The results suggest that feeding increasing levels of SBM improves G:F and CE. The energy value of SBM was estimated to be between 105% and 125% of corn, which is much greater than the NRC (2012) would indicate.

## Background

Soybean meal (SBM) is the primary plant-protein source for swine diets in the United States. The amino acid (AA) profile of SBM is well-balanced and complements the AA profile of grains such as corn and wheat, and these AA are highly digestible for pigs [1]. The energy content of SBM has been reported [1] as 3619 kcal/kg digestible energy (DE) and 3294 kcal/kg metabolizable energy (ME), which suggests that SBM has 105% and 97% of corn grain DE and ME values, respectively. More recently, swine nutritionists have adopted the net energy (NE) system due to the higher correlation to performance relative to DE or ME systems [2]. Using the NE system, SBM contains 2087 kcal/kg, which is only 78% of the corn energy value [1]. However, recent research shows improvements in gain-to-feed ratio (G:F) of pigs fed increasing levels of SBM [3, 4], which could indicate that the NRC [1] underestimates the NE of SBM.

Calorimetry trials to measure NE involve labor-intensive procedures that require highly specialized equipment. A practical approach conducted under field conditions to estimate energy values has gained acceptance among swine nutritionists. Feeding increasing amounts of an ingredient and using the differences in caloric efficiency (CE) to estimate the energy content of a test ingredient relative to a known ingredient, usually corn, has been reported by others and sometimes termed productive energy [5–9]. As the inclusion of the test ingredient increases, CE should not change if its energy estimate is accurate. Increases or decreases in CE indicate over- or underestimation of the energy content. Therefore, the objective of this study was to determine differences in growth performance of pigs fed increasing amounts of SBM and, by using changes in CE, to estimate SBM energy value relative to corn.

## Methods

The Kansas State University Institutional Animal Care and Use Committee approved the protocol used in these experiments.

### Diets and experimental design

Representative samples of corn grain, SBM, and distillers dried grains with solubles were submitted to the Agricultural Experimental Station Chemical Laboratories (University of Missouri-Columbia, Columbia, MO, USA) for determination of total AA content (method 982.30; AOAC International [10]) prior to diet formulation (Table 1) in Exp. 1. The total AA values were multiplied by NRC [1] standardized ileal digestible coefficients and used in diet formulation. Corn, SBM, and distillers dried grains were also analyzed (Ward Laboratories, Inc., Kearney, NE) for dry matter (method 935.29; AOAC International [10]), crude protein (method 990.03; AOAC International [10]), neutral detergent fiber (Ankom [11]), and ether extract (Ankom [12]). For Exp. 2, diets were formulated using NRC [1] nutrient loadings.

**Table 1:** Proximate analysis and total amino acid content of corn, distillers dried grains with solubles (DDGS), and soybean meal fed in Exp. 1, as-is basis^a,b^

Item, %	Corn	DDGS	Soybean meal
Dry matter	87.8	90.8	88.8
Crude protein	6.3	28.7	48.0
Neutral detergent fiber	7.0	27.9	5.4
Ether extract	3.6	8.8	1.1
Calcium	0.07	0.08	0.42
Phosphorus	0.23	0.88	0.64
Amino acids			
Alanine	0.45	1.86	2.06
Arginine	0.30	1.27	3.42
Aspartic acid	0.44	1.79	5.39
Cysteine	0.16	0.60	0.73
Glutamic acid	1.11	3.64	8.44
Glycine	0.26	1.11	2.00
Histidine	0.19	0.78	1.27
Isoleucine	0.24	1.09	2.33
Leucine	0.71	3.19	3.71
Lysine	0.25	1.08	3.09
Methionine	0.13	0.50	0.66
Phenylalanine	0.31	1.69	2.52
Proline	0.56	2.07	2.41
Serine	0.29	1.26	1.91
Threonine	0.23	1.10	1.81
Tryptophan	0.06	0.22	0.73
Tyrosine	0.18	1.03	1.66
Valine	0.31	1.45	2.44

^a^A representative sample of each ingredient was obtained, homogenized, and submitted to the Agricultural Experimental Station Chemical Laboratories (University of Missouri-Columbia, Columbia, MO, USA) for amino acid analysis and Ward Laboratories (Kearney, NE) for proximate analysis prior to diet formulation

^b^For Exp. 2, the NRC (2012) [1]amino acid values were used in diet formulation

There were 4 dietary treatments in Exp. 1 consisting of increasing amounts of SBM (21%, 27%, 33%, or 39% of the diet) with 23 replicates per treatment. In Exp. 2, there were 6 dietary treatments (17.5%, 22.0%, 26.5%, 31.0%, 35.5%, or 40.0% SBM) with 14 replicates per treatment. The increasing amounts of SBM were obtained by changing the inclusion of feed-grade AA and corn grain (Tables 2 and 3). Diets were formulated to meet or exceed the NRC (2012) [1] requirement estimates and were not balanced for NE. The NRC [1] NE value for SBM (2087 kcal/kg) and corn (2672 kcal/kg) were used in diet formulation. The NE value for DDGS was estimated as a function of the oil content based on Graham et al. [7] equation. Diets were provided in mash form. The energy value of SBM relative to corn was estimated based on CE, which was obtained by multiplying ADFI by kcal of NE per kg of diet and dividing by ADG. In order to obtain an energy estimate, the energy value of SBM was adjusted for the slope of CE to be zero.

**Table 2:** Ingredient composition of experimental diets, Exp. 1, as-fed basis

	Soybean meal, %			
	21	27	33	39
Ingredient, %				
Corn	60.07	54.68	49.21	43.70
Soybean meal	21.00	27.00	33.00	39.00
DDGS	15.00	15.00	15.00	15.00
Calcium carbonate	1.08	1.08	1.08	1.08
Monocalcium phosphate, 21.5% P	0.65	0.55	0.50	0.40
Sodium chloride	0.50	0.50	0.50	0.50
*L*-Lys HCl	0.643	0.456	0.255	0.053
*DL*-Met	0.225	0.170	0.110	0.045
*L*-Thr	0.295	0.215	0.135	0.040
*L*-Trp	0.095	0.060	0.020	–
*L*-Val	0.225	0.115	–	–
*L*-Ile	0.040	–	–	–
Vitamin trace-mineral premix^a^	0.150	0.150	0.150	0.150
Phytase^b^	0.050	0.050	0.050	0.050
Total	100	100	100	100
Calculated analysis				
SID amino acids, %				
Lys	1.30	1.30	1.30	1.30
Ile:Lys	55	61	69	78
Leu:Lys	112	124	137	149
Met:Lys	37	34	32	30
Met+Cys:Lys	57	57	57	57
Thr:Lys	65	65	65	65
Trp:Lys	22.1	22.1	22.0	23.4
Val:Lys	76	76	76	85
His:Lys	33	37	42	47
Net energy^c^, kcal/kg	2475	2437	2398	2362
Crude protein, %	19.2	21.3	23.4	25.6
Neutral detergent fiber, %	11.9	11.7	11.5	11.3
Calcium, %	0.69	0.70	0.72	0.74
STTD P, %	0.45	0.45	0.45	0.45
Analyzed values, %				
Dry matter	87.7	88.1	88.2	88.5
Crude protein	20.0	21.4	24.2	25.9
Neutral detergent fiber	9.2	9.8	9.8	9.3
Ether extract	2.9	3.0	2.8	2.6

^a^Provided per kg of premix: 5,344,543 IU vitamin A; 1,336,137 IU vitamin D; 100,211 IU vitamin E; 1,671 mg vitamin K; 21.4 mg vitamin B_12_; 29,061 mg niacin; 15,366 mg pantothenic acid; 4,008 mg riboflavin; 66.8 mg biotin; 668 mg folic acid; 1,202 mg vitamin B_6_; 73 g Zn from zinc sulfate; 67 g Fe from ferrous sulfate; 27 g Mn from manganese oxide; 10 g Cu from copper sulfate; 0.5 g I from calcium iodate; 0.2 g Se from sodium selenite

^b^Optiphos 2000 (Huvepharma, Inc., Peachtree City, GA, USA)

^c^Net energy values were obtained from the NRC (2012) [1]

**Table 3:** Ingredient composition of experimental diets, Exp. 2, as-fed basis

	Soybean meal, %					
	17.5	22.0	26.5	31.0	35.5	40.0
Ingredient, %						
Corn	62.69	58.78	54.86	50.90	46.98	43.07
Soybean meal	17.50	21.99	26.48	31.01	35.5	40.00
DDGS	15.00	15.00	15.00	15.00	15.00	15.00
Calcium carbonate	1.35	1.36	1.36	1.37	1.37	1.37
Monocalcium phosphate, 21.5% P	0.46	0.37	0.28	0.19	0.09	–
Sodium chloride	0.43	0.42	0.42	0.41	0.40	0.40
*L-*Lys sulfate	1.694	1.355	1.016	0.678	0.339	–
Met hydroxy analog	0.195	0.159	0.123	0.088	0.052	0.016
*L-*Thr	0.247	0.198	0.148	0.099	0.050	–
*L-*Trp	0.071	0.057	0.043	0.028	0.014	–
*L-*Val	0.147	0.117	0.088	0.059	0.030	–
*L-*Ile	0.061	0.049	0.037	0.024	0.012	–
Vitamin trace-mineral premix^a^	0.150	0.150	0.150	0.150	0.150	0.150
Total	100	100	100	100	100	100
Calculated analysis						
SID amino acids, %						
Lys	1.20	1.20	1.20	1.20	1.20	1.20
Ile:Lys	55	60	66	71	76	81
Leu:Lys	124	133	142	151	160	169
Met:Lys	37	36	35	34	33	32
Met+Cys:Lys	58	59	59	60	61	62
Thr:Lys	64	65	66	67	68	69
Trp:Lys	19.2	20.1	21.0	21.8	22.7	23.5
Val:Lys	70	74	77	81	85	88
His:Lys	34	38	42	45	49	52
Net energy, kcal/kg	2455	2433	2411	2388	2366	2344
Crude protein, %	18.9	20.5	22.1	23.7	25.3	26.9
Neutral detergent fiber, %	12.17	12.19	12.20	12.21	12.22	12.23
Calcium, %	0.69	0.69	0.69	0.69	0.69	0.69
STTD P, %	0.38	0.38	0.37	0.36	0.36	0.35
Analyzed values, %						
Dry matter	85.7	86.0	85.9	86.2	86.9	86.8
Crude protein	17.2	19.2	20.2	22.7	23.7	25.6
Crude fiber	2.2	2.4	2.7	3.1	3.0	3.2
Ether extract	3.1	3.1	3.1	3.1	3.3	3.3

^a^Provided per kg of premix: 1,653,468 IU vitamin A; 661,387 IU vitamin D; 17,637 IU vitamin E; 1323 mg vitamin K; 13.2 mg vitamin B_12_; 19,842 mg niacin; 11,023 mg pantothenic acid; 3307 mg riboflavin; 499,899 FTU phytase; 73 g Zn from zinc sulfate; 67 g Fe from ferrous sulfate; 27 g Mn from manganese oxide; 10 g Cu from copper sulfate; 0.5 g I from calcium iodate; 0.2 g Se from sodium selenite

### Animals and housing

Experiment 1 was conducted at New Horizon Farms Nursery Research (Pipestone, MN). A total of 2233 pigs (PIC 337 × 1050, Hendersonville, TN) were placed in 92 pens containing 20 to 27 mixed gender pigs and used in a 21-d trial. Each pen (3.7 m × 2.3 m) had plastic floors and was equipped with a six-hole stainless steel dry feeder and a pan waterer. Experiment 2 was conducted at the JBS Research Facility (Tipton, MO, USA). A total of 3796 pigs (PIC 359 × C40, Hendersonville, TN, USA) were placed in 84 pens with 37 to 43 pigs per pen. Each pen (6.9 m × 3.6 m) had fully slated floors and was equipped with a 4-hole stainless steel wet-dry feeder and a nipple waterer.

Pigs were weaned at approximately 21 d of age, placed in pens based on initial body weight (BW), and fed common diets until the start of the experiments. Pens of pigs were blocked by BW (initial BW = 11.0 kg in Exp. 1 and 17.6 kg in Exp. 2, respectively) and allotted to 1 of 4 or 6 treatments in Exp. 1 and 2, respectively, in a randomized complete block design. Pens of pigs were weighed, and feed disappearance was measured weekly to determine average daily gain (ADG), average daily feed intake (ADFI), G:F, and CE. Culls and mortality were recorded daily.

### Chemical analysis

Representative diet samples were obtained from each treatment and stored at − 20 °C until analysis. Samples were analyzed for dry matter (method 935.29; AOAC International [10]), crude protein (method 990.03; AOAC International [10]), calcium (method 985.01; AOAC International [10]), phosphorus (method 985.01; AOAC International [10]), neutral detergent fiber (Ankom [11]), and ether extract (Ankom [12]).

### Statistical analysis

Data were analyzed as a randomized complete block design with initial BW as the blocking factor. Single degree-of-freedom contrasts were constructed to test the linear and quadratic effects of increasing SBM. Block was included as a random effect and treatment as a fixed effect. Pen was considered the experimental unit. Data were analyzed using the GLIMMIX procedure of SAS 9.4 (SAS Institute Inc., Cary, NC, USA). Data distribution was assessed using visual inspection of histograms prior to statistical analysis. Results were considered significant at *P* ≤ 0.05 and a tendency at 0.05 < *P* ≤ 0.10.

## Results and discussion

### Chemical analysis

The analyzed amino acid profiles of corn, DDGS, and SBM used in Exp. 1 were, in general, within the expected values (Table 1). Soybean meal and DDGS had a similar amino acid composition to NRC [1] values, whereas AA in corn were slightly lower than NRC [1] estimates, especially for Met and Leu. The chemical analysis of diets was consistent with formulated values (Tables 2 and 3).

### Growth performance and energy estimate

In Exp. 1, there was a tendency (linear, *P* = 0.092) for a decrease in ADFI as dietary SBM increased (Table 4). Pigs fed diets with increasing SBM had a tendency (quadratic, *P* = 0.090) for an improvement in ADG up to 33% SBM, followed by a decrease in ADG when 39% SBM was fed. The changes in ADG and ADFI resulted in a tendency (quadratic, *P* = 0.069) for an improvement in G:F up to 33% SBM. There was an improvement (linear, *P* = 0.001; quadratic, *P* = 0.063) in CE with increasing SBM. There was no evidence (*P* > 0.10) for difference in cull and mortality rate.

**Table 4:** Effects of increasing soybean meal inclusion on growth performance and caloric efficiency of pigs, Exp. 1^a^

	Soybean meal, %				SEM	Probability, *P*	
Item	21	27	33	39		Linear	Quadratic
BW, kg							
d 0	11.0	11.0	11.0	11.0	0.15	< 0.894	< 0.993
d 21	22.3	22.3	22.4	22.0	0.28	< 0.263	< 0.180
d 0 to 21							
ADG, g	537	537	543	524	7.3	< 0.207	< 0.090
ADFI, g	824	822	815	804	11.7	< 0.092	< 0.579
G:F, g/kg	652	653	667	653	5.1	< 0.390	< 0.069
CE, kcal/kg gain	3801	3738	3600	3623	28.8	< 0.001	< 0.063
Culls and mortality, %	0.72	0.36	0.36	0.36	0.356	< 0.457	< 0.596

^a^A total of 2233 pigs (initially 11.0 kg) were used in a 21-d study with 20 to 27 pigs per pen and 23 replicates per treatment

In Exp. 2, there was a reduction (linear, *P* = 0.001) in ADFI as SBM increased. Pigs fed increasing SBM had a tendency (linear, *P* = 0.065) for reduced ADG (Table 5). However, the differences were relatively small and did not result in differences (*P* ≥ 0.27) in final BW. There was an improvement (linear, *P* = 0.001) in G:F and CE as SBM increased. There was a reduction (linear, *P* = 0.050) in cull and mortality rate as SBM increased.

**Table 5:** Effects of increasing soybean meal inclusion on growth performance and caloric efficiency of pigs, Exp. 2^a^

	Soybean meal, %						SEM	Probability, *P*	
Item	17.5	22.0	26.5	31.0	35.5	40.0		Linear	Quadratic
BW, kg									
d 0	17.5	17.6	17.5	17.6	17.6	17.6	0.20	< 0.801	< 0.997
d 22	35.6	35.8	35.5	35.4	35.5	35.4	0.28	< 0.272	< 0.987
d 0 to 22									
ADG, g	820	825	818	809	812	809	7.4	< 0.065	< 0.922
ADFI, g	1500	1509	1473	1424	1415	1401	18.1	< 0.001	< 0.957
G:F, g/kg	547	548	556	568	574	578	5.4	< 0.001	< 0.893
CE, kcal/kg gain	4491	4450	4342	4203	4126	4055	43.6	< 0.001	< 0.955
Culls and mortality, %	0.58	0.89	0.71	0.43	0.14	0.14	0.447	< 0.050	< 0.377

^a^A total of 3796 pigs (initially 17.6 kg) were used in a 22-d study with 37 to 43 pigs per pen and 14 replicates per treatment

A considerable amount of research has been conducted to evaluate the effects of SBM on growth performance of pigs. It is well known that the addition of SBM should be restricted in the diet immediately after weaning due to a hypersensitivity reaction [13, 14], but the restriction is not necessary after initial exposure. Nevertheless, feeding SBM is usually limited due to the high cost compared to diets formulated with high amounts of feed-grade AA as a replacement of intact protein sources. However, some research suggests feeding diets with higher amounts of SBM could prove beneficial, especially for health challenged pigs. Johnston et al. [15] fed 21% or 32% SBM to grow-finish pigs that were infected with porcine reproductive and respiratory syndrome (PRRS) and observed that pigs fed 32% SBM had improved ADG and G:F compared with those fed 21% SBM. Similarly, Rocha et al. [16] observed improvements in G:F of nursery pigs inoculated with PRRS virus and fed 22.5% SBM compared to 12.5% SBM. Rochell et al. [17] found that PRRS positive nursery pigs had improved ADG when fed 29% SBM compared to 17.5% SBM. Conversely, Cemin et al. [4] fed 27% or 35% SBM to PRRS negative nursery pigs and observed improvements in ADG and G:F as SBM increased. Moran et al. [3] conducted two trials evaluating increasing amounts of SBM for nursery pigs. In the first trial, pigs were PRRS negative and the authors observed a consistent improvement in G:F. However, the results were not repeated in the subsequent study when pigs originated from a PRRS positive sow farm performance.

Interestingly, Moran et al. [3] found a reduction in the percentage of pigs removed for medical treatment from 11.1% to 8.4% as SBM increased. This observation is in agreement to our finding in Exp. 2, where increasing SBM linearly reduced cull rate. The benefits of SBM on growth performance, especially for health challenged pigs, have also been hypothesized to be driven by bioactive components such as isoflavones and saponins, which have anti-inflammatory, antioxidant, and anti-viral properties [18, 19]. However, the available research is inconsistent regarding the effects of isoflavones on growth performance of pigs. Kuhn et al. [20] compared SBM and soy protein concentrate, an ingredient with markedly lower isoflavones relative to SBM, in a wean-to-finish study and observed higher plasma isoflavones in pigs fed SBM but no evidence for differences in growth performance. Greiner et al. [21, 22] evaluated increasing dietary isoflavones and observed improvements in performance of PRRS positive pigs, mostly during periods of peak viremia. Smith et al. [19] fed diets based on soy protein concentrate or enzyme-treated SBM with or without isoflavones and observed changes in activation of the adaptive immune system, although no impact on growth performance was observed.

There was a tendency for a quadratic response in ADG with increasing SBM in Exp. 1, with a decrease observed in ADG of pigs fed the highest SBM inclusion. Similarly, in Exp. 2 there was a slight reduction in ADG with increasing SBM. However, the differences between treatments were relatively small and did not result in statistical differences in final BW. Feed intake was also negatively affected by high levels of SBM. The reason for the negative response of high SBM inclusion on ADFI and ADG is unclear. Although the available literature generally does not agree with this finding, as the majority of studies [3, 4, 15–17] found no change or improvements in ADG with increasing SBM, the current experiment evaluated higher SBM levels than most of the previous research. It could be hypothesized that the high level of crude protein in the diet with 39% SBM provided excess nitrogen which needs to be metabolized and excreted by the animal [23]. The excess nitrogen represents an energy cost that may ultimately translate to decreased growth performance. Moreover, the Leu:Lys increased with increasing levels of SBM. It is well known that excessive Leu leads to reduction in feed intake [24]; however, recent research suggests that the negative impact may be counteracted by concomitant increases in Ile, Val, and Trp [25], as observed in our experimental diets.

Improvements in feed efficiency with increasing amounts of SBM seem to be more consistently reported in the literature and agree with our findings. Energy is the most expensive component of any swine diet, thus it is critical to accurately determine the energy value of feedstuffs. Direct measurement of NE is a procedure that requires highly specialized equipment. Therefore, the estimation of the energy value of a test ingredient based on CE relative to a known ingredient such as corn grain is suggested as a practical approach, and is sometimes termed productive energy [5–9]. Besides the practical advantage, the estimates using CE conducted under field conditions may be more predictive of growth performance than other energy values. The diets used in our experiments were formulated using the NRC [1] NE value for SBM and were not balanced for energy; thus, as SBM increased, dietary NE decreased. The resulting dietary NE values ranged from 2475 to 2362 kcal/kg in Exp. 1 and 2455 to 2344 kcal/kg in Exp. 2. Therefore, if the NE of SBM provided by the NRC [1] was accurate, G:F should become worse as SBM level increased in the diet. However, the improvement in CE observed in the current experiments suggest that the NE value of SBM was underestimated. The NRC [1] NE value for SBM is 2087 kcal/kg or 78% of corn NE. Our findings from Exp. 1 based on CE suggest that the energy value of SBM is 105.4% of corn grain energy or 2816 kcal/kg NE. It is important to note that, while CE response was significantly linear (*P* = 0.001), there was also a tendency (*P* = 0.065) for a quadratic response. Therefore, it could be hypothesized that the CE value of 39% SBM treatment should not be considered for estimating energy because slope-ratio assays should only include the linear portion of the response [26]. By removing the 39% SBM diet and using the linear portion of the dataset results in an energy estimate of 121.1% of corn grain energy or 3236 kcal/kg NE. A similar response was observed in Exp. 2, where energy value of SBM was estimated as 124.7% of corn grain energy or 3332 kcal/kg. The energy estimates of both experiments are greater than the NRC [1] NE value, which may be driven by Noblet et al. [27] equations too severely penalizing the NE content of high crude protein ingredients. However, it is important to note the using CE to estimate the energy value of an ingredient as a ratio to corn has limitations. This approach assumes that the NE values of corn are accurate and does not account for changes in body composition, which can influence the CE response as leaner pigs are more efficient [28]. Using indirect calorimetry, Li et al. [29] found that a NE of 2709 kcal/kg for SBM, which is 101.4% of NRC [1] corn NE and significantly greater than the NRC [1] SBM NE value.

Another important consideration is that the responses in performance could have been driven by underestimation of the AA requirements relative to Lys. Our diets were formulated to meet or exceed the NRC [1] requirement estimates; nevertheless, if any of these estimates is not accurate, by increasing the inclusion of SBM we could have potentially corrected an AA deficiency. However, most of the AA ratios were well above that recommended by the NRC [1], thus the responses to SBM are unlikely to be driven by changes in AA ratios.

## Conclusion

Nursery pigs fed diets with increasing amounts of SBM presented inconsistent responses in ADG, but G:F and CE were improved in both experiments. The results of the current study suggest that the energy value of SBM may be estimated to range between 105% and 125% of corn energy, or 2816 and 3332 kcal/kg NE, which indicates that the NRC [1] potentially underestimates the SBM NE value. This has important ramifications as it increases the value of SBM in diet formulation. However, it is unclear if the benefit of higher inclusion of SBM is entirely driven by energy or if another underlying mechanism, potentially involving intrinsic SBM components such as isoflavones, could be partially responsible for the response observed in this study.

## Data Availability

Data availability upon request to the corresponding author.

## Abbreviations

AA: Amino acid
ADFI: Average daily feed intake
ADG: Average daily gain
BW: Body weight
CE: Caloric efficiency
DDGS: Distillers dried grains with solubles
DE: Digestible energy
G:F: Gain-to-feed ratio
ME: Metabolizable energy
NE: Net energy
PRRS: Porcine reproductive and respiratory syndrome
SBM: Soybean meal
SID: Standardized ileal digestible
STTD: Standardized total tract digestible
